# Evaluation of the Antihyperuricemic Activity of Phytochemicals from *Davallia formosana* by Enzyme Assay and Hyperuricemic Mice Model

**DOI:** 10.1155/2014/873607

**Published:** 2014-05-04

**Authors:** Chen-Yu Chen, Chi-Chang Huang, Keng-Chang Tsai, Wei-Jan Huang, Wen-Ching Huang, Yu-Chen Hsu, Feng-Lin Hsu

**Affiliations:** ^1^School of Pharmacy, College of Pharmacy, Taipei Medical University, Taipei 110, Taiwan; ^2^Graduate Institute of Pharmacognosy, Taipei Medical University, Taipei 110, Taiwan; ^3^Graduate Institute of Sports Science, National Taiwan Sport University, Taoyuan 333, Taiwan; ^4^National Research Institute of Chinese Medicine, Taipei 112, Taiwan; ^5^Graduate Institute of Athletics and Coaching Science, National Taiwan Sport University, Taoyuan 333, Taiwan

## Abstract

Abnormal serum urate levels are recognized as a critical factor in the progression of several chronic diseases. To evaluate the antihyperuricemic effect of *Davallia formosana*, the inhibitory activities of 15 isolated phytochemicals, including five novel compounds of 6,8-dihydroxychromone-7-*C*-**β**-d-glucopyranoside (**1**), 6,8,3′,4′-tetrahydroxyflavanone-7-*C*-**β**-d-glucopyranoside (**2**), 6,8,4′-trihydroxyflavanone-7-*C*-**β**-d-glucopyranoside (**3**), 8-(2-pyrrolidinone-5-yl)-catechin-3-*O*-**β**-d-allopyranoside (**4**), and epiphyllocoumarin-3-*O*-**β**-d-allopyranoside (**5**), were examined against xanthine oxidase (XOD) and in a potassium oxonate-(PTO-) induced acute hyperuricemic mice model. The results indicated that compounds **3** and **5** significantly inhibited XOD activity *in vitro* and reduced serum uric acid levels *in vivo*. This is the first report providing new insights into the antihyperuricemic activities of flavonoid glycosides which can possibly be developed into potential hypouricemic agents.

## 1. Introduction

Hyperuricemia means high levels of uric acid in the blood, a condition considered to be closely associated with increased risks for developing gout, cardiovascular diseases, hypertension, and metabolic syndrome [1, 2]. Xanthine oxidase (XOD) is an important enzyme responsible for the catabolism of purines in humans; it oxidizes hypoxanthine into xanthine and then further forms uric acid [3, 4]. Allopurinol is currently the most effective XOD inhibitor, which is used for treating hyperuricemia and gout by reducing circulating levels of uric acid and vascular oxidative stress [5]. However, serious side effects include skin rashes and allergic reactions that occur in some clinical patients [6, 7].

Recently, several naturally occurring compounds were reported to inhibit XOD activity [8–10]. In particular, plant phenolic compounds, such as phenolic acids and flavonoids, exhibit strong antioxidant activities via scavenging free radicals. Moreover, many studies also indicated that both types of compounds obviously inhibited XOD activity [11–13].* Davallia formosana* is a popular herbal medicine used to treat osteoporosis [14]. Several flavan-3-ols, triterpenoids, proanthocyanidins, and mericprocyanidins were isolated from the rhizome of* D. formosana* [15, 16]. Our preliminary studies revealed that the crude extract of* D. formosana* rhizomes could inhibit XOD activity. Therefore, in this study, we investigated the constituents of* D. formosana* and their antihyperuricemic effects. An* in vitro* XOD-inhibitory assay and* in vivo* potassium oxonate- (PTO-) induced acute hyperuricemic mouse model were used to evaluate the uric acid-lowering effects of compounds isolated from* D. formosana*.

## 2. Materials and Methods

### 2.1. Chemicals and Reagents

PTO, allopurinol, sodium pyrophosphate, xanthine, and XOD were purchased from Sigma Chemical (St. Louis, MO). The solvents used for column chromatography, including methanol,* i*-BuOH,* n*-BuOH, dichloromethane (CH_2_Cl_2_), chloroform (CHCl_3_),* n*-hexane, ethyl acetate (EtOAc), and acetone, were purchased from Merck (Darmstadt, Germany).

### 2.2. General Experimental Procedures

^1^H- and ^13^C-NMR spectra were obtained in the Bruker AM-500 spectrometer using corresponding solvents as internal standards. The optical rotation was measured on a Jasco DIP-1020 digital polarimeter. Electrospray ionization mass was determined on a VG platform electrospray mass spectrometer. Column chromatography was performed using Sephadex LH-20 (20~100 *μ*m, Pharmacia Fine Chemicals, China), MCI-gel CHP 20P (75~150 *μ*m, Mitsubishi Chemical Industries, Japan), Cosmosil C_18_-OPN (75 *μ*m, Nacalai Tesque), and silica gel 60 (70~230 mesh, Merck). Thin layer chromatography (TLC) was conducted on silica gel plates (60 F-254, Merck), with a 10% sulfuric acid solution as the visualizing agent on heating.

### 2.3. Plant Material

*Davallia formosana* was collected in Kaohsiung, Taiwan, in July 2010. It was authenticated by Dr. Hsien-Chang Chang (Division of Pharmacognosy, National Laboratories of Food and Drugs, Department of Health, Taiwan). A voucher specimen was deposited at the Department of Medicinal Chemistry, College of Pharmacy, Taipei Medical University, Taipei, Taiwan.

### 2.4. Extraction, Isolation, and Identification

Dry rhizomes of* D. formosana* (50 kg) were extracted with 80% ethanol at room temperature. The total ethanolic extract was evaporated in a vacuum. The residue (11 kg) was successively partitioned with* n*-hexane, EtOAc, and* n*-BuOH. The* n*-BuOH fraction (1.4 kg) was chromatographed on a Diaion HP20 column (25 × 120 cm) with a step gradient system (H_2_O to MeOH, 1 : 0~0 : 1,50 L) to yield two fractions. The major fraction, DF-B (390 g), was further subjected to a Sephadex LH-20 column eluted with H_2_O/MeOH (1 : 0~0 : 1) to afford four subfractions (DFB-1~4). The DFB-4 subfraction (263.7 g) was chromatographed over an MCI CHP20 (H_2_O/MeOH 1 : 0~0 : 1) to obtain four subfractions (DFB-41~44). The DFB-43 subfraction (40.5 g) was subsequently purified on a Sephadex LH 20 (acetone) and silica gel with a CHCl_3_-MeOH gradient to give** 2** (62.0 mg),** 3** (33.0 mg),** 5** (115.0 mg), (-)-epicatech-3-*O*-*β*-d-(2′′-*O*-vanillyl)-allopyranoside (**7**, 760.0 mg), and (-)-epicatech-3-*O*-*β*-d-(3′′-*O*-vanillyl)-allopyranoside (**8**, 230.0 mg). (-)-Epicatech-3-*O*-*β*-d-allopyranoside (**6**, 30.0 g) was obtained by recrystallization (in acetone) of DFB-41 (37.3 g). The DFB-3 subfraction (30.3 g) was subjected to MCI CHP20 (H_2_O to MeOH, 1 : 0~0 : 1) and Sephadex LH-20 (H_2_O) column chromatography to yield** 4** (5.0 mg), eriodictyol-8-C-*β*-d-glucopyranoside (**9**, 1.1 g), davallioside A (**10**, 31.0 mg), davallioside B (**11**, 20.0 mg), and caffeic acid-4-*O*-*β*-d-glucopyranoside (**12**, 210.0 mg). The DFB-2 fraction (62.4 g) was subjected to MCI CHP20 column chromatography with H_2_O/MeOH gradient system to give eight subfractions (DFB-21~28). Subfraction DFB-23 (17.7 g) from H_2_O eluent was purified by MCI CHP20 gel and Sephadex LH-20 column to yield** 1** (537.0 mg) and* p*-coumaric acid-4-*O*-*β*-d-glucopyranoside (**13**, 92.7 mg).

Protocatehuic acid (**14**, 730.0 mg), 4-hydroxy-3,5-dimethylbenzoic acid (**15**, 97.3 mg), vanillic acid (**16**, 86.0 mg), 4-hydroxy-3-aminobenzoic acid (**17**, 60.0 mg), and (-)-epicatechin (**18**, 202.0 mg) were obtained from EtOAc fraction (170.0 g). Davallic acid (**19**, 6.0 g) and *β*-stiosterol (**20**, 644.0 mg) were isolated from* n*-hexane fraction (114.0 g).

*6,8-Dihydroxychromone-7-C-*β*-* d *-glucopyranoside* (**1**). White amorphous powder; [*α*]_D_^24^ − 91.2° (*c* = 0.5, MeOH); IR (KBr) *v*_max⁡_: 3332, 1650, 1631, 1568 cm^−1^; UV (MeOH) *λ*_max⁡_: 210, 258, and 298 nm; HR-ESI-MS *m*/*z*: 339.0717 [M-H]^−^ (calcd. for C_15_H_15_O_9_, 339.0716).

*6,8,3*′*,4*′*-Tetrahydroxyflavanone-7-C-*β*-* d *-glucopyranoside* (**2**). White amorphous powder; [*α*]_D_^24^ – 43.2° (*c* = 1.0, MeOH); IR (KBr) *v*_max⁡_: 3250, 1652, 1584, 1539 cm^−1^; UV (MeOH) *λ*_max⁡_: 205.0 and 288.5 nm; HR-ESI-MS *m*/*z*: 449.1087 [M-H]^−^ (calcd. for C_21_H_21_O_11_, 449.1084).

*6,8,4*′*-Trihydroxyflavanone-7-C-*β*-* d *-glucopyranoside* (**3**). White amorphous powder; [*α*]_D_^24^ – 21.8° (*c* = 0.5, MeOH); IR (KBr) *v*_max⁡_: 3315, 1615, 1518 cm^−1^; UV (MeOH) *λ*_max⁡_: 226.0 and 290.0 nm; HR-ESI-MS *m*/*z*: 433.1142 [M-H]^−^ (calcd. for C_21_H_21_O_10_, 433.1135).

*8-(2-Pyrrolidinone-5-yl)-catechin-3-O-*β*-* d *-allopyranoside *(**4**). Yellow amorphous solid; [*α*]_D_^24^ + 9.7° (*c* = 1.0, MeOH); IR (KBr) *v*_max⁡_: 3334, 1646, 1611 cm^−1^; UV (MeOH) *λ*_max⁡_: 209 and 280 nm; HR-ESI-MS *m*/*z*: 534.1617 [M-H]^−^ (calcd. for C_25_H_28_NO_12_, 534.1612).

*Epiphyllocoumarin-3-O-*β*-* d *-allopyranoside* (**5**). White amorphous powder; [*α*]_D_^24^ – 96.7° (*c* = 0.2, MeOH); IR (KBr) *v*_max⁡_: 3314, 1707, 1613, 1573, 1531 cm^−1^; UV (MeOH) *λ*_max⁡_: 208 and 334 nm; HR-ESI-MS *m*/*z*: 503.1203 [M-H]^−^ (calcd. for C_24_H_23_O_12_, 503.1190).

*Acid hydrolysis of* **5**. Compound** 5** (10 mg) was hydrolyzed with 2 N HCl in aqueous MeOH (5 mL) for 4 h, and the product was further extracted with EtOAc. The H_2_O layer was passed through silica gel eluted with CHCl_3_/MeOH/H_2_O (4 : 2 : 0.1) to give a sugar residue. The sugar was analyzed by silica gel TLC [*i*-PrOH-Me_2_CO-H_2_O (5 : 3 : 1), *R*_*f*_ 0.55] and compared to authentic samples.

### 2.5. Determination of XOD-Inhibitory Activity

The inhibitory effect on XOD was determined spectrophotometrically [17]. The reaction mixture consisted of 100 *μ*L of 50 mM potassium phosphate buffer (pH 7.5), 50 *μ*L of 1.5 mM xanthine, 10 *μ*L of sample solution dissolved in dimethyl sulfoxide (DMSO), and 25 *μ*L XOD (0.05 U). The absorption increments at a UV absorbance of 295 nm indicated the formation of uric acid. All determinations were performed in triplicate. Pure compounds and allopurinol for the XO inhibitory activity assays were examined at concentrations of 0, 25, 50, and 100 *μ*M, respectively. The inhibitory activity of XOD was assessed as the inhibitory percent (%) = (1 − b/a) × 100, where “a” is the change in absorbance per minute without the sample, and “b” is the change in absorbance per minute with the sample.

### 2.6. Hypouricemic Effects Examined in Mice with PTO-Induced Hyperuricemia

Six-week-old male ICR mice with body weights of about 30.0 g were purchased from BioLASCO (A Charles River Licensee Corp., Yilan, Taiwan). Before the experiments, mice were raised for 1 week to allow them to acclimate to the environment and diet. All mice were given a standard laboratory diet (no. 5001; PMI Nutrition International, Brentwood, MO) and distilled water* ad libitum* and kept on a 12 h light/dark cycle at 22 ± 2°C. This study was conducted according to institutional guidelines and was approved by the Institutional Animal Care and Utilization Committee (IACUC) of National Taiwan Sport University, Taoyuan, Taiwan. This study was approved by the IACUC ethics committee under protocol IACUC-10004.

Test animals were intraperitoneally (*i.p.*) injected with phosphate-buffered saline (PBS) containing 200 mg/kg of PTO 1 h before administration of test samples adapted from recent studies [18, 19]. Mice were randomly divided into six groups for treatment (*n* = 6): (1) a vehicle group; (2) PTO group; (3) PTO + allopurinol (AP) group; (4) PTO +** 2** group; (5) PTO +** 3** group; (6) PTO +** 5** group. For the comparative study, the same dosages of 100 mmol/kg of AP (13.6 mg/kg), compound** 2** (45.0 mg/kg), compound** 3** (43.4 mg/kg), and compound** 5** (50.4 mg/kg) were delivered* i.p*. at 1 h after PTO administration. Blood samples were centrifuged at 1400 ×g and 4°C for 15 min, and the level of serum uric acid was determined by a commercial kit from Randox Laboratories (UK).

### 2.7. Xanthine Oxidase Molecular Docking

Models of compound** 5** in complex with xanthine oxidase were generated through docking compound** 5** to the active site of the X-ray crystal structure of bovine xanthine oxidase (PDB id: 3B9J) (Figure 5). In order to predict the position of compound** 5** in the active site, we implemented the docking program (GOLD Genetic Optimization for Ligand Docking) (Cambridge Crystallographic Data Center (CCDC), version 3.2) with the Goldscore scoring function. Before docking, the substrate 2-hydroxy-6-methylpurine and all water molecules were removed. The 3D structure of compound** 5** was generated and optimized by energy minimization using Discovery Studio v.3.5 (Accelrys Software Inc., USA). GOLD was used to dock compound** 5** into the proteins with the flexible docking option turned on. Initially, 500 independent genetic algorithm cycles of computation were carried out with ligand torsion angles varying between −180° and 180°. The search efficiency was set at 200% to ensure the most exhaustive search for the docking conformational space. All other parameters were kept the same as the default settings. Finally, from the 500 docking conformations of compound** 5**, the top one with the highest GOLD fitness score was chosen to explore the “inhibitor-bond” conformations in the xanthine oxidase active site using Goldscore within the GOLD program. The molecular models of compound** 5** were displayed using the PyMOL software (http://www.pymol.org).

### 2.8. Statistical Analysis

All data are expressed as the mean ± standard error of the mean (SEM). A one-way analysis of variance (ANOVA) was performed with Duncan's post hoc test for multisample testing. *P* < 0.05 was considered statistically significant.

## 3. Results and Discussion

### 3.1. Structure Elucidation

**Figure 1:**
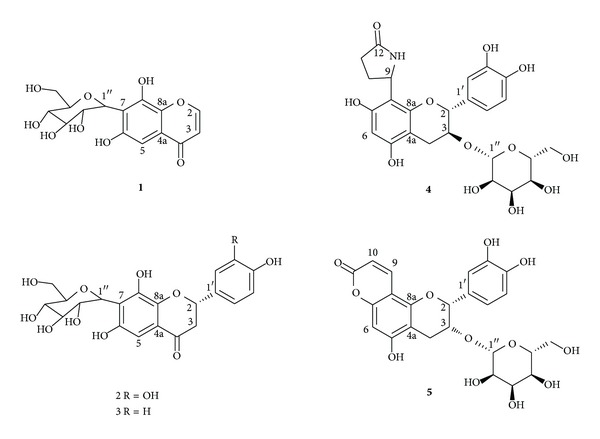
Chemical structures of new compounds** 1**–**5** isolated from the rhizome of* Davallia formosana*.

The rhizome of* D. formosana* was extracted with 80% ethanol and repeatedly chromatographed to obtain 20 compounds. Five new compounds (Figure 1) together with 15 known compounds, including seven flavonoids, six phenolics, and two triterpenoids, were elucidated based on the physical and spectral data.

Compound** 1** was obtained as a white amorphous powder. The HR-ESI-MS analysis agreed with the molecular formula C_15_H_16_O_9_ (*m*/*z* = 339.0717 [M-H]^−^). The ^1^H and ^13^C-NMR data showed typical signals of a flavone nucleus and a glucose unit. Resonances of the flavone moiety were assigned at *δ* 8.02 (1H, d, *J* = 6.1 Hz, H-2), 6.27 (1H, d, *J* = 6.1 Hz, H-3), and *δ* 6.50 (1H, s, H-5). The deshielding of the chemical shifts at *δ* 163.3 and 160.5 indicated a 6,8-dihydroxyl substitution. Glucose signals were determined by ^1^H-^1^H COSY and HMQC spectra. The site of the glucose linkage to the flavone was considered to be C-7 from the HMBC experiment. These results suggested that the structure of compound** 1** was 6,8-dihydroxychromone-7-*C*-*β*-d-glucopyranoside.

Compound** 2** was isolated as a white amorphous powder with optical rotation [*α*]_D_^24^ – 43.2°. The negative HR-ESI-MS exhibited quasimolecular ion peaks at *m*/*z* 449.1087 [M-H]^−^. ^1^H-NMR and ^1^H-^1^H COSY spectra showed typical flavanone structural features at *δ* 5.91 (1H, s, H-5), 5.34 (1H, dd, *J* = 11.9, 2.8 Hz, H-2), 2.70 (1H, dd, *J* = 17.9, 3.1 Hz, H-3), and 3.03–3.16 (1H, m, H-3). The coupling constant of 17.9 Hz observed at *δ* 2.70 indicated that the C-2 substituted aryl group was equatorial. The ABX-type resonance at *δ* 6.85 (1H, s, H-2′), 6.74 (1H, d, *J* = 8.5 Hz, H-6′), and 6.68 (1H, d, *J* = 8.5 Hz, H-5′) indicated the presence of 1,3,4-trisubstitutions in the B-ring. Additionally, the ^1^H-NMR spectrum exhibited signals at *δ* 3.03–4.46 for a sugar moiety. The COSY and HMQC spectra indicated that the sugar moiety was a glucopyranose. The *β*-configuration of the glucose moiety was determined by the coupling constant of an anomeric proton (*J* = 9.8 Hz). The resonances of C-6 and C-8 were significantly shifted downfield to *δ* 166.3 and 162.0, and the HMBC spectrum showed a correlation between the aromatic proton (*δ* 5.91) and carbonyl carbon (*δ* 196.9). This evidence indicated that hydroxyl groups were substituted at C-6, 8. Moreover, the HMBC experiment further showed the three-bond correlation between the anomeric proton H-1′′ (*δ* 4.46) and C-6, 8 (*δ* 166.3, 162.0), suggesting that the glucose was joined to the A-ring of the aglycone through a* C*-glycosidic linkage at C-7. Therefore, the structure of** 2** was characterized as 6,8,3′,4′-tetrahydroxyflavanone-7-*C*-*β*-d-glucopyranoside.

The molecular formula of compound** 3** was established as C_21_H_22_O_10_ on the basis of HR-ESI-MS data (*m*/*z* 433.1142 for [M-H]^−^). The ^1^H-NMR spectrum of** 3** was similar to that of** 2**, except that the AX-type resonance at *δ* 6.81 and 7.31 (each 2H, *J* = 8.5 Hz) replaced the ABX-type coupling pattern of compound** 2**. Six carbon signals at *δ* 82.9, 80.8, 75.5, 72.9, 72.2, and 63.2 were assigned as a glucopyranose. The orientation of glucose was confirmed to be the *β*-configuration according to the coupling constant of the anomeric proton (*J* = 9.8 Hz). The HMBC correlation between glucopyranose H-1′′ and aglycone C-7 suggested that glucose was substituted at C-7 of the aglycone. The molecular weight of** 3** lost 16 units compared to that of** 2**, which supported compound** 3** having lost a hydroxyl group at position C-5′. Accordingly, the structure of compound** 3** was assigned as 6,8,4′-trihydroxyflavanone-7-*C*-*β*-d-glucopyranoside.

**Figure 2:**
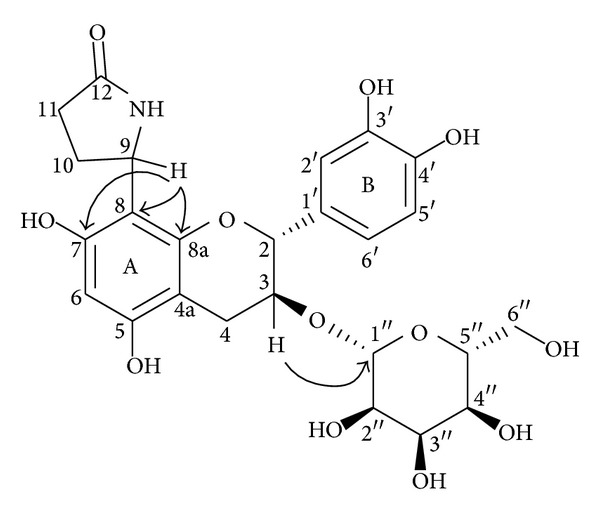
HMBC correlations of compound** 4**.

Compound** 4** was isolated as a yellow amorphous solid. The molecular formula was deduced to be C_25_H_29_NO_12_ from HR-ESI-MS at *m*/*z* 534.1628 [M-H]^−^. According to the ^1^H and ^13^C-NMR data, the compound showed a characteristic catechin structure feature at *δ* 5.07 (1H, d, *J* = 5.5 Hz, H-2), 4.32 (1H, m, H-3), and 2.70 (2H, d, *J* = 5.5 Hz, H-4). The *J*_2,3_ coupling constant (*J* = 5.5 Hz) confirmed the* trans*-arrangement of H-2 and H-3. Analysis of ^1^H-^1^H COSY and HMQC spectra, the deshielding of the carbonyl signal at *δ* 181.6, a tertiary carbon signal at *δ* 50.3 (C-11), and two methylene signals at *δ* 32.3, 27.0 indicated that a *γ*-lactam group was contained in the structure. This result was further demonstrated by IR absorption at 1646 cm^−1^. The long-rang correlation between the signal at *δ* 5.37 (1H, dd, *J* = 9.5, 4.6 Hz, H-11) and C-7, 8, 8a suggested that the *γ*-lactam ring was attached to C-8 (Figure 2). The allopyranose moiety was determined by ^1^H-^1^H COSY and HMBC spectra. Additionally, the HMBC correlation between H-3 and C-1′′ demonstrated that the allopyranose residue was linked to C-3 of the aglycone. These results indicated that compound** 4** was similar to davallioside A and B [20]. Therefore, compound** 4** was a catechin-3-*O*-*β*-d-allopyranoside with a *γ*-lactam substitution at C-8, and it was determined by 8-(2-pyrrolidinone-5-yl)-catechin-3-*O*-*β*-d-allopyranoside.

Compound** 5** was a white amorphous powder, and the molecular formula was determined to be C_24_H_24_O_12_ (*m*/*z* 503.1203 for [M-H]^−^) by the HR-ESI-MS spectrum. The ^1^H and ^13^C-NMR data (Supplementary Tables  1 and  2 available online at http://dx.doi.org/10.1155/2014/873607) showed that compound** 5** was comprised of three units, including a flavan-3-ol, a sugar unit, and an *α*, *β*-unsaturated carbonyl group. The relative configurations at H-2 and H-3 were assigned as a* cis*-orientation based on the coupling constant of signals at *δ* 5.32 (1H, br.s, H-2) and 4.44 (1H, d, *J* = 2.4 Hz, H-3). The methylene protons at *δ* 2.81 (1H, dd, *J* = 16.2, 4.3 Hz) and 2.65 (1H, dd, *J* = 16.2, 5.2 Hz) of H-4 indicated a flavan moiety structure of compound** 5**. Resonances of four aromatic protons included a typical ABX-type spin system of the 1,3,4-trisubstituted benzene ring [*δ* 6.95 (brs, H-2′), 6.76 (d, *J* = 8.5 Hz, H-5′), and 6.66 (d, *J* = 8.5 Hz, H-6′)] and one singlet at *δ* 6.38 attributed to the A-ring of flavan-3-ol. The HMBC correlations between the olefinic proton *δ* 7.99 (H-10) and C-8 (*δ* 101.8), as well as *δ* 6.10 (H-9) and C-7 (*δ* 154.7), indicated an *α*, *β*-unsaturated carbonyl group linked to the C-7, 8 of A-ring. These results revealed that these resonances were consistent with the pyrone ring of a coumarin entity [21]. Acid hydrolysis of** 5** yielded epiphyllocoumarin [22] and allopyranose. The *β*-configuration of the sugar moiety was determined by the coupling constant of the anomeric proton *δ* 4.51 (1H, d, *J* = 7.3 Hz, H-1′). The HMBC correlation of H-3 to C-1′ demonstrated that the allopyranose residue was linked to C-3 of the aglycone. Therefore,** 5** was characterized as epiphyllocoumarin-3-*O*-*β*-d-allopyranoside.

Fifteen known compounds, including (-)-epicatech-3-*O*-*β*-d-allopyranoside (**6**) [23], (-)-epicatech-3-*O*-*β*-d-(2′′-*O*-vanillyl)-allopyranoside (**7**) [16], (-)-epicatech-3-*O*-*β*-d-(3′′-*O*-vanillyl)-allopyranoside (**8**) [16], eriodictyol-8-*C*-*β*-d-glucopyranoside (**9**) [24], davallioside A (**10**) [20], davallioside B (**11**) [20], caffeic acid-4-*O*-*β*-d-glucopyranoside (**12**) [25], *p*-coumaric acid-4-*O*-*β*-d-glucopyranoside (**13**) [25], protocatehuic acid (**14**) [26], 4-hydroxy-3,5-dimethylbenzoic acid (**15**) [27], vanillic acid (**16**) [28], 4-hydroxy-3-aminobenzoic acid (**17**) [29], (-)-epicatechin (**18**) [30], davallic acid (**19**) [15], and *β*-sitosterol (**20**) [31], were identified by comparison with values in the literature. Among them, compounds** 9**~**17** were first isolated from* D. formosana*.

### 3.2. The XOD-Inhibitory Activity of Phytochemicals from* D. formosana*

**Figure 3:**
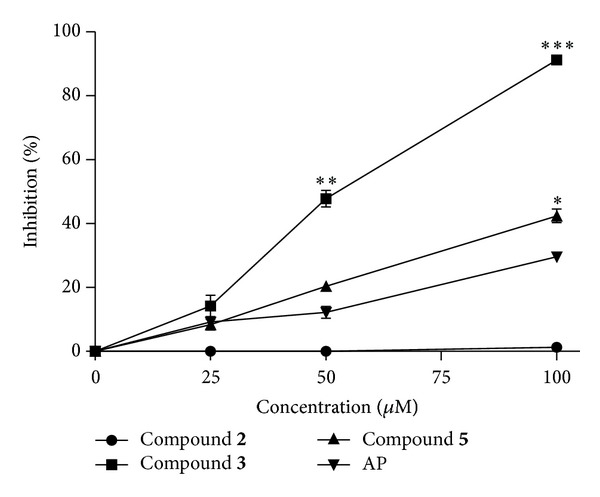
The xanthine oxidase- (XOD-) inhibitory activity of phytochemicals from* Davallia formosana*. AP, allopurinol (positive control);** 2**, 6,8,3′,4′-tetrahydroxyflavanone-7-*C*-*β*-d-glucopyranoside;** 3**, 6,8,4′-trihydroxyflavanone-7-*C*-*β*-d-glucopyranosid;** 5**, epiphyllocoumarin-3-*O*-*β*-d-allopyranoside. Each value represents the mean ± SEM of three replicates. *P < 0.05, **P < 0.01, and ***P < 0.001, compared to the positive control.

The XOD-inhibitory activities of all pure compounds isolated from* D. formosana* were examined with effects comparable to that of allopurinol (Supplementary Table  3), a clinically used inhibitor, at the same concentration. Compounds** 3** and** 5** significantly inhibited XOD activity in dose-dependent manners, compared to allopurinol (Figure 3). The IC_50_ of compounds** 3** and** 5** was 57.4 and 124.0 *μ*M, respectively, whereas there was no detectable effect for compound** 2**.

### 3.3. *In Vivo* Hypouricemic Effect Determined in Mice with PTO-Induced Hyperuricemia

**Figure 4:**
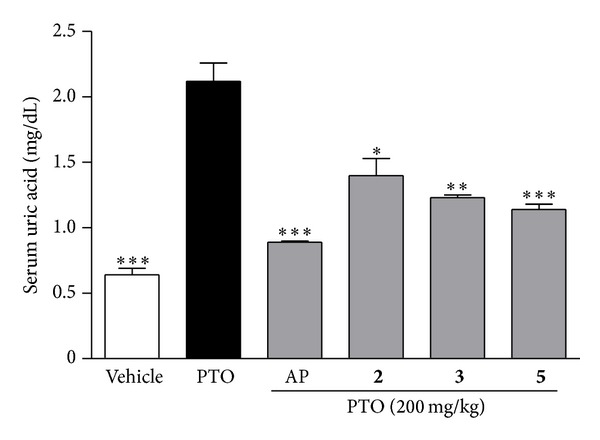
The uric acid-lowering effects of compounds** 2**,** 3**, and** 5** from* Davallia formosana* on mice with potassium oxonate- (PTO-) induced hyperuricemia. The results are presented as the mean ± SEM (*n* = 6). *P < 0.005, **P < 0.001, and ***P < 0.0001, compared to the PTO-treated group.

To further confirm the capabilities of compounds** 2**,** 3**, and** 5** to reduce the uric acid level* in vivo*, a PTO-induced hyperuricemia mice model was investigated. After 3 h of PTO treatment, the level of serum uric acid had increased to 12 ± 0.14 mg/dL. As shown in Figure 4, PTO-induced serum uric acid levels were reduced by the three test compounds, as well as the reference (allopurinol). At the same concentration (100 mmol/kg), compounds** 2**,** 3**,** 5**, and allopurinol significantly reduced the level of serum uric acid by 33.9%, 41.7%, 46.0%, and 58.1%, respectively, compared to the PTO group (*P* < 0.005). XOD is an important purine metabolic enzyme, which is a significant target for developing antihyperuricemic drugs. The* in vitro* and* in vivo* results suggested that inhibition of XOD activity played an important role in the antihyperuricemic effects of compounds** 3** and** 5**.

### 3.4. Computational Docking Studies of Compound **5**

In addition to its uric acid-lowering activity* in vivo*, compound** 5** also inhibits xanthine oxidase activity* in vitro*. We were interested in visualizing the effects of compound** 5** on XOD in order to gain insights into the observed activities. Many studies have shown that flavonoids can inhibit XOD activity via hydrogen bonding and hydrophobic interaction with key amino acid residues on XOD-catalyzed sites such as Arg880, Phe914, Phe1009, and Thr1010 [32]. To determine the preferred positions of binding sites on XOD for compound** 5**, the 3D model of interaction was analyzed by docking using bovine milk XOD (PDB id: 3B9J)[33].

**Figure 5:**
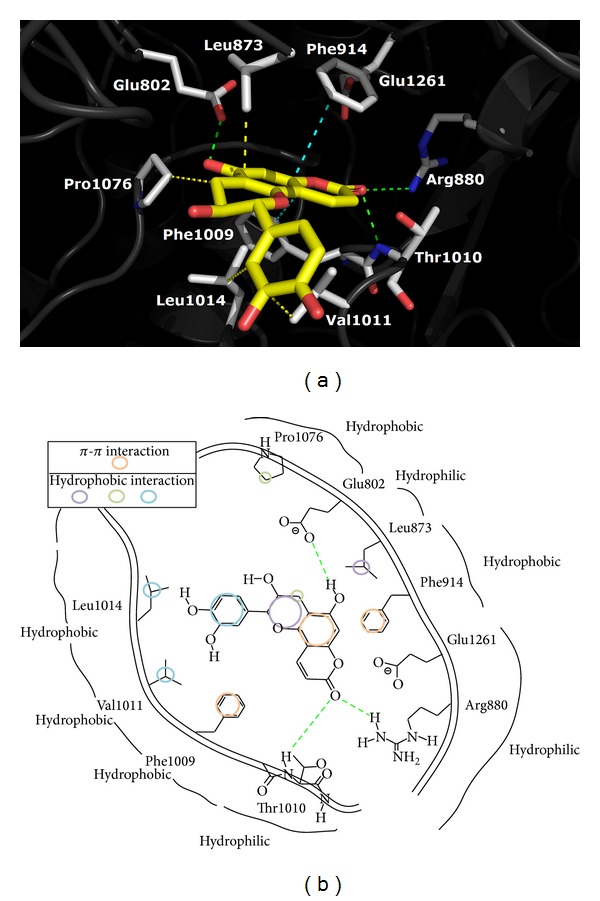
Predicted binding mode of compound** 5** docked into the active site of xanthine oxidase. The top and down pictures of each panel display the 3D and 2D structural docking patterns, respectively. The nitrogen and oxygen atoms are shown in dark blue and red colors, respectively. The hydrogen bond formation and the electrostatic interaction between compound** 5** (yellow) and the amino acid residues (gray) of XOD are shown in green and light blue dashed lines, respectively.

As shown in Figure 5, the carbonyl group on the benzopyranone forms hydrogen bonds with the active sites, including Arg880 and Thr1010. Furthermore, the 5-hydroxyl group of compound** 5** forms hydrogen bonds with Glu802. The docking results show again that the A-ring of compound** 5** is sandwiched between Phe914 and Phe1009 and participates in the formation of aromatic interactions (*π*-*π* effects) with the two phenylalanines. In addition, three hydrophobic interactions were observed involving the methylene and Pro1076, the 3′,4′-dihydroxyphenyl moiety and Leu1014, and Val1011. It can be seen that binding residues are the same among [34], suggesting that Arg880 and Thr1010 might be of significance for the selective inhibition of XOD by compound** 5**.

This study obtained five new compounds from* D. formosana*, and two of them exhibited potent antihyperuricemic activity. Accordingly, our results can provide the scientific basis for development of antihyperuricemic drugs.

## Supplementary Material

Supplementary Table S1: ^1^H-NMR (500 MHz) spectral data of compounds 1-5.
Supplementary Table S2: ^13^C-NMR (125 MHz) spectral data of compounds 1-5.
Supplementary Table S3: XOD-inhibitory activities of 20 isolated phytochemicals from *D. formosana*
