# Supplementary material for: Evaluation of the Antihyperuricemic Activity of Phytochemicals from Davallia formosana by Enzyme Assay and Hyperuricemic Mice Model
Source: Evid Based Complement Alternat Med. 2014 May 4;2014:873607. doi: 10.1155/2014/873607 (PMC4026843; doi:10.1155/2014/873607)
Supplement: Supplementary file 1 — Supplementary Table S1: 1H-NMR (500 MHz) spectral data of compounds 1-5. Supplementary Table S2: 13C-NMR (125 MHz) spectral data of compounds 1-5. Supplementary Table S3: XOD-inhibitory activities of 20 isolated phytochemicals from D. formosana [file 873607.f1.doc]

**Supplementary Material**

**Supplementary TABLE 1: ^1^H-NMR (500 MHz) spectral data of compounds** 1-5

| Position | **1^a^** | **2^b^** | **3^a^** | **4^a^** | **5^a^** |
| --- | --- | --- | --- | --- | --- |
| **2** | 8.02 *d* (5.5) | 5.34 *dd* (11.9, 2.8) | 5.35 *dd* (12.5, 2.8) | 5.07 *d* (5.5) | 5.32 *br.s* |
| **3** | 6.27 *d* (5.5) | 2.70 *dd* (17.1, 3.1) | 2.7 *dd* (17.1, 3.1) | 4.32 *m* | 4.44 *d* (2.4) |
|  |  | 3.03-3.16 *m* | 3.13 *dd* (17.1, 12.8) |  |  |
| **4** |  |  |  | 2.7 *d* (5.5) | 2.65 *dd* (16.2, 5.2)  2.81 *dd* (16.5, 4.2) |
| **5** | 6.50 *s* | 5.91 *s* | 5.96 *s* |  |  |
| **6** |  |  |  | 5.97 *s* | 6.38 *s* |
| **9** |  |  |  | 5.37 *dd* (9.5, 4.6) | 7.99 *d* (9.2) |
| **10** |  |  |  | 2.22-2.31 *m* | 6.10 *d* (9.2) |
| **11** |  |  |  | 2.34-2.46 *m* |  |
| **2'** |  | 6.85 *s* | 6.81 *d* (8.5) | 6.79 *s* | 6.95 *br.s* |
| **3'** |  |  | 7.31 *d* (8.5) |  |  |
| **5'** |  | 6.68 *d* (8.5) | 7.31 *d* (8.5) | 6.67 *d* (8.0) | 6.66 *d* (8.0) |
| **6'** |  | 6.74 *d* (8.5) | 6.81 *d* (8.5) | 6.70 *d* (8.0) | 6.76 *d* (8.0) |
| **1''** | 4.87 *d* (9.8) | 4.46 *d* (9.8) | 4.78 *d* (9.8) | 4.84 *overlap* | 4.51 *d* (7.3) |
| **2''** | 4.21 *dd* (9.5, 4.4) | 3.96 *t* (9.2) | 4.07-4.16 *m* | 3.23 *dd* (7.6, 2.7) | 3.08 *td* (7.2, 3) |
| **3''** | 3.44-3.55 *m* | 3.03-3.30 *m* | 3.41-3.49 *m* | 4.01 *br.s* | 3.78 *d* (3) |
| **4''** | 3.44-3.55 *m* | 3.03-3.30 *m* | 3.41-3.49 *m* | 3.45 *dd* (9.5, 2.7) | 3.16 *m* |
| **5''** | 3.44-3.55 *m* | 3.03-3.30 *m* | 3.41-3.49 *m* | 3.61-3.68 *m* | 3.47 *m* |
| **6''** | 3.84-3.90 *m*  3.73-3.79 *m* | 3.43-3.50 *m*  3.64 *d* (11.6) | 3.85 *dd* (11.9, 1.5)  3.71 *dd* (12.2, 5.5) | 3.83 *m*  3.61-3.68 *m* | 3.40 *dt* (11.7, 6)  3.65 *m* |

a) Values in MeOH-*d*_4_. b) Values in DMSO-*d*_6_

**Supplementary TABLE 2: ^13^C-NMR (125 MHz) spectral data of compounds 1-5**

| **Position** |  | | **1^a^** | **2^b^** | **3^a^** | **4^a^** | **5^a^** |
| --- | --- | --- | --- | --- | --- | --- | --- |
| 2 | | 157.6 | | 78.7 | 80.5 | 81.0 | 78.3 |
| 3 | | 110.3 | | 42.5 | 44.2 | 74.6 | 74.8 |
| 4 | | 182.8 | | 196.9 | 198.1 | 24.3 | 23.9 |
| 4a | | 105.2 | | 102.0 | 103.6 | 100.3 | 104.6 |
| 5 | | 94.6 | | 95.2 | 96.7 | 157.0 | 160.3 |
| 6 | | 163.34 | | 166.3 | 167.6 | 96.4 | 94.8 |
| 7 | | 107.5 | | 106.3 | 106.3 | 156.4 | 154.7 |
| 8 | | 160.5 | | 162.0 | 159.4 | 107.9 | 101.8 |
| 8a | | 158.0 | | 163.6 | 164.6 | 155.2 | 151.8 |
| 9 | |  | |  |  | 50.3 | 139.5 |
| 10 | |  | |  |  | 27.0 | 110.0 |
| 11 | |  | |  |  | 32.3 | 161.1 |
| 12 | |  | |  |  | 181.6 |  |
| 1' | |  | | 129.9 | 129.3 | 132.3 | 129.0 |
| 2' | |  | | 114.8 | 116.7 | 116.3 | 115.4 |
| 3' | |  | | 145.7 | 129.3 | 145.0 | 144.9 |
| 4' | |  | | 146.2 | 131.3 | 146.1 | 145.2 |
| 5' | |  | | 115.8 | 129.3 | 115.1 | 116.0 |
| 6' | |  | | 118.4 | 116.7 | 119.6 | 118.9 |
| 1'' | | 73.5 | | 73.4 | 75.5 | 100.6 | 99.7 |
| 2'' | | 80.9 | | 82.0 | 82.9 | 73.1 | 71.0 |
| 3'' | | 78.4 | | 79.5 | 80.7 | 74.6 | 72.0 |
| 4'' | | 70.7 | | 71.1 | 72.9 | 69.0 | 68.1 |
| 5'' | | 70.0 | | 70.7 | 72.2 | 75.6 | 74.8 |
| 6'' | | 61.2 | | 62.0 | 63.2 | 63.3 | 62.1 |

a) Values in MeOH-*d*_4_. b) Values in DMSO-*d*_6_

**Supplementary TABLE 3:** XOD-inhibitory activities of 20 isolated phytochemicals from *D. formosana*

| Compounds | IC_50_ (μM) |
| --- | --- |
| 6,8-dihydroxychromone-7-*C*-β-D-glucopyranoside (**1**) | >500 |
| 6,8,3',4'-tetrahydroxyflavanone-7-*C*-β-D-glucopyranoside (**2**) | >500 |
| 6,8,4'-trihydroxyflavanone-7-*C*-β-D-glucopyranoside (**3**) | 57.4±1.9 |
| 8-(2-pyrrolidinone-5-yl)-catechin-3-*O*-β-D-allopyranoside (**4**) | >500 |
| epiphyllocoumarin-3-*O*-β-D-allopyranoside (**5**) | 124.0±1.3 |
| (-)-epicatechin (**6**) | >500 |
| (-)-epicatechin-3-*O*-β-D-allopyranoside (**7**) | >500 |
| (-)-epicatechin-3-*O*-β-D-(2"-*O*-vanillyl)-allopyranoside (**8**) | >500 |
| (-)-epicatechin-3-*O*-β-D-(3"-*O*-vanillyl)-allopyranoside (**9**) | >500 |
| eriodictyol-8-*C*-β-D-glucopyranoside (**10**) | >500 |
| davallioside A (**11**) | >500 |
| davallioside B (**12**) | >500 |
| caffeic acid-4-*O*-β-D-glucopyranoside (**13**) | >500 |
| *p*-coumaric acid-4-*O*-β-D-glucopyranoside (**14**) | >500 |
| protocatehuic acid (**15**) | >500 |
| 4-hydroxy-3-aminobenzoic acid (**16**) | >1000 |
| vanillic acid (**17**) | >1000 |
| 4-hydroxy-3,5-dimethylbenzoic acid (**18**) | >1000 |
| davallic acid (**19**) | >1000 |
| β-sitosterol (**20**) | >1000 |
| Allopurinol | 202.3±0.8 |
